# Comparison of results on the use of extended criteria liver doners for transplants in Espírito Santo

**DOI:** 10.1590/0100-6991e-20202492

**Published:** 2021-04-24

**Authors:** ANTONIO CARLOS LUGON FERREIRA-JR, GUSTAVO PEIXOTO SOARES MIGUEL, IARA MOSCON, ISAAC WALKER ABREU, JULIETE BOREL DE OLIVEIRA SILVA AGUIAR, THALES RIBEIRO DOS SANTOS VECCI

**Affiliations:** 1 - Meridional Hospital, Transplant Center - Cariacica - ES - Brasil; 2 - Federal University of Espírito Santo, Surgycal Clinic - Vitória - ES - Brasil

**Keywords:** Liver Transplantation, Hepatic Cirrhosis, Donator Selection, Tissue and Organ Procurement, Transplante de Fígado, Cirrose Hepática, Seleção do Doador, Obtenção de Tecidos e Órgãos

## Abstract

**Introduction:**

liver Transplantation is currently the treatment of choice for several terminal liver diseases. Despite the increase in performed transplants, the waiting lists continue to increase. In order to expand the supply of organs, transplantation teams have started to use previously rejected livers for transplants because of an increased risk of unfavorable outcomes*.*

**Objective:**

to evaluate the use of livers of expanded criterion donators.

**Methods:**

retrospective study of medical records. The livers were classified as normal or expanded criteria. The groups were divided in low and high MELD. A multivariate analysis was performed through logistic regression*.*

**Results:**

there was no statistical difference regarding early, late and global mortality between the groups. Decreased survival was observed in patients with high MELD (higher or equal to 20) when they received grafts from expanded criterion donators. The association between the occurrence of cardiorespiratory arrest and presence of elevated total bilirubin in donators was associated with higher mortality rates in expanded criterion livers.

**Conclusion:**

the overall results are similar, but expanded criteria liver donators was associated with higher mortality in patients with high MELD.

## RESUMO

**Introdução:**

o Transplante Hepático é atualmente o tratamento de escolha para diversas doenças terminais do fígado. Apesar do aumento de transplantes realizados, as filas de espera continuam aumentando. Com a finalidade de ampliar a oferta de órgãos, as equipes transplantadoras passaram a utilizar fígados até então rejeitados para transplantes devido ao risco aumentado de desfechos desfavoráveis.

**Objetivo:**

avaliar utilização de fígados de doadores de critério expandido.

**Métodos:**

estudo retrospectivo por meio de análise de prontuários. Classificaram-se os fígados utilizados em padrão ou critério expandido. Os grupos foram subdivididos em MELD baixo e alto. Foi realizada análise multivariada por regressão logística.

**Resultados:**

não houve diferença estatística com relação à mortalidade precoce, tardia e global entre os grupos. Observou-se menor sobrevida em pacientes com MELD alto (maior ou igual a 20) quando receberam enxertos de doadores de critério expandido. Foi observada a associação entre ocorrência de parada cardiorrespiratória e presença de bilirrubina total elevada no doador com maiores taxas de mortalidade em receptores de fígados de critério expandido.

**Conclusão:**

os resultados globais são semelhantes, porém o uso de fígado de doadores de critério expandido esteve associado a maior mortalidade em pacientes com MELD alto.

## INTRODUCTION

Orthotopic liver transplantation (OLT) has developed in recent decades and is currently the treatment of choice for terminal liver diseases¹. The evolution of OLT techniques led to development in hepatobiliary surgery, trauma surgery, and intensive support to the surgical patient, contributing to surgical learning². New perspectives have emerged for the management of patients with advanced liver diseases, making these procedures routine in several centers. However, with better results and definitions of criteria and indications for OLT, a new obstacle arises: the disproportion between patients on the waiting list and organs supply.

In Brazil, liver transplantation is regulated by the Ministry of Health Ordinance Nº 2,600, of October 21, 2009, with the objective of updating, perfecting, and standardizing the operation of the National Transplant System, thus determining which indications and situations characterize priority^3^. Ordinance Nº 1,160, of May 29, 2006 modified the criterion for the distribution of brain death donor livers to the severity of the recipient’s clinical condition, adopting the MELD system - model for end stage liver disease - for adults and adolescents over 12 years old, and the PELD - pediatric end stage liver disease - for children under 12 years old. Serum dosages of total bilirubin, creatinine, and international standardized ratio of prothrombin activity (INR) are used to calculate MELD, and serum dosages of total bilirubin, albumin, and INR, to compute PELD^4^. In 2019, sodium was included as a criterion for calculating MELD-Na.

Transplantation teams started using liver grafts that had been previously rejected, the so-called marginal donors, expanded criteria livers or expanded criteria donors (ECD)^8^, to expand organ supply. Many studies have been collaborating and showing acceptable results using expanded criteria livers, with the objective of reducing the waiting time in the transplant queues. Many efforts have been made to define criteria, parameters, and cutoff points, but there is still no precise definition by the transplant community. Several risk factors, such as advanced donor age, prolonged cold ischemia time, prolonged hospital stay, hypotension, hepatic steatosis, and high sodium levels are the most studied factors related with increased risk^8^ ^-^ ^16^. Studies carefully try to demonstrate how these risk factors can be combined to maintain satisfactory and acceptable results^10^.

## METHODS

We conducted a retrospective study, with analysis of medical records of patients in brain death who had their organs donated, at the Central Notification, Collection, and Distribution of Organs of the Brazilian state of Espirito Santo - CNCDO/ES. In particular, we considered the livers offered for transplantation, harvested, and implanted by the liver transplantation team of the Hospital Meridional, from July 2007 to August 2013, according to the inclusion and exclusion criteria mentioned below.

During this period, we operated 142 patients. We excluded one case of pediatric interventional transplant and another 31 cases, due to incomplete data. After initial screening, 110 patients remained to be evaluated.

In the sample, 88 patients were male (80%) and 22 female (20%). The mean age was 51.2 years (SD 11.3), with the youngest transplanted patient being 14 years old, and the oldest, 69. The patients’ average MELD was 17 (SD 6.7), ranging between six and 41. Regarding the previous history, 46 patients (41.8%) had reported alcohol use, 22 (20%) had chronic hepatitis B, and 26 (23.6%), hepatitis C. Hepatocellular carcinoma (HCC) was present in 34 cases. Other causes were responsible for 29 transplants (26.4%), among them, fulminant hepatitis, primary and secondary biliary cirrhosis, Budd-Chiari syndrome, metastasis of carcinoid tumors, non-alcoholic steatohepatitis (NASH), and cryptogenic. 

The criterion for assessing and distributing the grafts in two groups was similar to that used by Bacchella et al., initially proposed by Briceño et al. We assigned 1 point to age > 60 years, period of orotracheal intubation > 4 days, cold ischemia time > 13 hours, hepatic macrosteatosis > 30%, total bilirubin > 2.0 mg/dl, alanine aminotransferase (ALT) > 170 U/l, and aspartate aminotransferase > 140 U/l. We assigned 2 points to use of vasopressor medication (any dosage of noradrenaline or dobutamine) and serum sodium dosage > 155 mEq/L. We considered a liver with an expanded criteria when its score was greater than or equal to 3^17^. We excluded Ischemia time (> 13h) and hepatic steatosis from the analysis, since fatty livers are discarded due to the absence of histopathological examination during harvesting. As for ischemia time, the organ is only accepted if the estimated time to implant is less than 12 hours.

The statistical analysis started by characterizing the sample. Concerning the recipient, we recorded the patient’s age at the time of transplantation and preoperative MELD. As for the donor, we recorded age, sex, laboratory variables used to distribute the groups, and cause of brain death. Regarding the characteristics of the harvest-to-implant process, we analyzed cold ischemia time, solution used for perfusion and organ preservation, hot ischemia time, and organ harvest site. 

We obtained two groups, 56 patients who received grafts from expanded criteria donors (ECD) and 54 patients receiving standard grafts (SG). We performed a descriptive analysis of the recipients with mean, standard deviation, minimum and maximum values referring to age and MELD classification, as shown in Table 1. We also subdivided the SG and ECD groups according to high and low MELD. As for sex in the SG group, 44 were male (81.5%) and 10 female (18.5%); in the ECD group, 44 were male (78.6%) and 10 were female (21.4%).

**Table 1:** Characterization of recipients by age and MELD, and the variables concerning surgery and used to classify the quality of the graft.

	SG				ECD				General			
	Min	Max	Mean	SD	Min	Max	Mean	SD	Min	Max	Mean	SD
Age (years) Recipient	14	67	51.3	11.8	15	69	51.2	10.9	14	69	51.2	11.3
MELD Recipient	6	31	16	5	6	41	17	6	6	41	17	6.7
Age (years) Donor	8	59	30.6	11.8	10	69	31.4	14.9	8	69	31.0	14.7
ICU-OTI (days) Donor	0	12	2.4	2.0	1	30	5.3	4.9	0	30	3.9	4.0
AST (U/L) Donor	8	153	66.1	41.6	16	841	121.3	146.0	8	841	97.4	116.0
ALT (U/L) Donor	9	138	38.6	28.1	10	375	77.4	77.2	8	375	60.2	63.3
TB (mg/dL) Donor	0.02	2.34	0.8	0.5	0.16	3.9	0.9	0.8	0.02	3.9	0.85	0.71
Na (mEq/L) Donor	130	173	145.5	8.3	130	199	159.5	12.5	130	199	153.4	12.9
VAD Donor	Yes	39 72.2%	No	15 27.8%	Yes	53 94.6%	No	3 5.4%	Yes	92 83.6%	No	18 16.4%
WI (min)	16	45	30.8	7.4	15	60	32.0	8.5	15	60	31.4	8.0
CI (min)	255	945	482.1	148.1	300	1060	568.2	187.5	255	1060	526.0	174.0

SG - Standard graft donor; ECD - Expanded criteria donor; SD - Standard deviation; ICU - Intensive care unit; OTI - Orotracheal intubation; AST - Aspartate amino transferase; ALT - Alanine aminotransferase; TB - Total bilirubin; Na - Sodium; VAD - Vasoactive drug; WI - Warm ischemia; CI - Cold ischemia.

In the evaluation of donors, we performed an analysis of age, mean time of intubation, AST, ALT, total bilirubin, serum sodium (Na), use of vasoactive medication (VAD), distributed in the SG and ECD groups, as shown in Table 1. Regarding sex, in the SG group 77.8% were male and 22.2% female, while in the ECD 78.6% were male, and 21.4%, female. 

During the organ donation process, from the event that caused brain death to the moment of liver uptake, seven donors (13%) experienced cardiopulmonary arrest (CPA) in the SG group, undergoing resuscitation measures and later stabilization of the cardiological condition. Meanwhile, in the ECD group, three donors (5.4%) had CPA complications, being treated in the same way as in the SG group (Table 2).

**Table 2:** Occurrence of CPA in the donor, cause of brain death, site of organ harvesting, preservation solution used for liver perfusion, and early, late, and global survival according to low and high MELD.

			SG		ECD		General	
			n	%	n	%	n	%
CPA	No		47	87.0	53	94.6	100	90.9
	Yes		7	13.0	3	5.4	10	9.1
	Trauma		28	65.1	31	66.0	59	53.6
Cause of brain death	Stroke		13	30.2	14	29.7	27	24.5
	Others		2	4.7	2	4.3	4	3.6
	Greater Vitória		29	56.9	26	46.4	55	51.4
Cause of brain death	The state countryside		20	39.2	22	39.3	42	39.3
	Another state		2	3.9	8	14.3	10	9.3
	Celsior		18	56.3	18	62.1	36	59.0
Preservation solution	IGL 1		14	46.7	9	31.0	23	37.7
	SPS		0	0.0	2	6.9	2	3.3
	MELD ≥ 20	No	38	95	37	90.2	75	92.6
Death between 0 and 30 days		Yes	2	5	4	9.8	6	7.4
	MELD ≥ 20	No	13	92.8	11	73.3	24	82.7
		Yes	1	7.2	4	26.7	5	17.3
	MELD < 20	No	33	86.8	34	91.9	67	89.4
Death between 31 and 90 days		Yes	5	13.2	3	8.1	8	10.6
	MELD ≥ 20	No	11	84.6	8	72.7	19	79.2
		Yes	2	15.4	3	27.3	5	20.8
	MELD < 20	No	33	82.5	44	80.5	77	84.6
		Yes	7	17.5	7	19.5	14	15.4
Death between 0 and 90 days	MELD ≥ 20	No	11	78.6	8	53.3	19	65.5
		Yes	3	21.4	7	46.7	10	34.5

SG - Standard graft donor; ECD - Expanded criteria donor; CPA - Cardiopulmonary arrest.

Among the causes of death in the SG group, the most common was traumatic brain injury (TBI), encompassing the various causes (accidents and gunshot or stab wounds), followed by stroke in both groups, as described in Table 2.

The most frequent sites for the harvesting of livers classified as SG were in greater Vitória, capital of the state, with 29 procurements (56.9%), 20 (39.2%) in the state countryside, and two (3.9%) in other states. Similarly, in the ECD group the most frequent donor sites were in greater Vitória, with 26 harvests (46.4%), 22 (39.3%) in the state countryside and eight (14.3%) in others States (Table 2).

Table 1 describes the variables referring to the operation, such as the time of cold ischemia and warm ischemia and preservation solutions used for liver perfusion.

We performed statistical analysis to verify the cause and effect relationship of age and clinical variables with deaths in less than 30 days, between 31 and 90 days, and from the time of surgery to 90 days. We used multiple logistic regression with backward eliminations (removes variables from the regression equation that are not influencing factors for the model, as per the Wald significance test)^18^. We also presented the Odds Ratio (OR) and the pseudo-r^2^, which is similar to the coefficient of determination (proportion of explanation of the dependent variable as a function of the independent ones) of the linear regression^19^.

The level of significance adopted was 5%, with a 95% confidence interval, and the software used in the analyzes was the IBM SPSS Statistics, version 21.

## RESULTS

The survival observed in the SG group in the early period (0 to 30 days) was 94.4%, in the late period (31 to 90 days), 86.3%, the overall survival (0 to 90 days) being 81.5%. In the ECD group, survival was 85.7% in the early period and 87.5% in the late one, the overall survival being 75% (Figure 2). The survival of the entire early sample was 90%, the late being 84.9%, and the overall, 78.2%.

**Figure 1:**
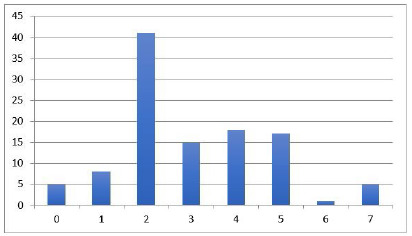
Distribution of grafts according to the severity score.

**Figure 2:**
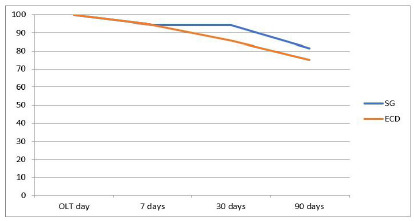
Survival in the SG and ECD groups.

**Figure 3:**
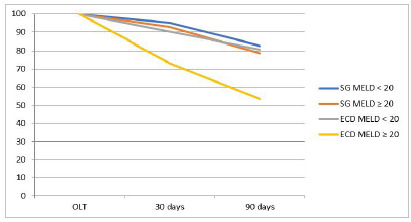
Survival in the SG and ECD groups according to MELD, less than 20 and greater than or equal to 20.

We subdivided survival according to high and low preoperative MELD. In the SG group, patients with MELD up to 19 displayed early survival of 95%, late of 86.8%, and overall of 82.5%. In those with MELD greater than or equal to 20, early survival was 92.8%, late was 84.6%, and overall, 82.5%. In the ECD group, patients with MELD up to 19 had early survival of 90.2%, late of 91.9%, and overall of 80.5%. In those with MELD greater than or equal to 20, early survival was 73.3%, late survival was 72.7%, and overall was 53.3% (Table 2 and Figure 3).

As for the frequency of primary non-functioning, in the SG group it was four cases (7.4%), three of whom died early (less than 7 days); the remaining patient underwent retransplant, but died within 53 days after the operation. In the ECD group, there were also four cases, 7.1%, of whom tow died prematurely and two others underwent retransplant, in which one progressed to early death and the other survived longer than 90 days.

Regarding retransplantation (RTX) in both groups, there were two RTX (3.7%) in the SG group, one due to late hepatic artery thrombosis (after 30 days) that evolved well, and the other due to primary non-functioning (PNF), who later died, thus displaying a 50% survival. In the ECD group, there were also RTX, both by PNF, with one favorable outcome and one early death, also representing a 50% survival rate.

There was no statistically significant clinical variable in the SG group, whereas in the ECD group, elevated total bilirubin (TB) was statistically significant, increasing the risk of death by 3.06 times (p = 0.019). The occurrence of donor CPA was associated with an increase in the risk of death by 17.89 times (p = 0.043), in addition, MELD greater than or equal to 20 was associated with a death risk increase of 6.99 times (p = 0.046) (Table 3). Logistic regression for the late period in the SG group indicated an association between prolonged cold ischemia and an increased risk of death. However, in the ECD group, the increase was negligible, 0.99 times (p = 0.049). In addition, the presence of elevated preoperative MELD (≥ 20) was associated with an increase in mortality risk by 10.94 (p = 0.035) (Table 3).

**Table 3:** Multiple logistic regression for the periods 0 to 30 days, 31 to 90 days, and 0 to 90 days.

		Pseudo r^2^	p value	OR	95% CI for OR	
					Bottom	Higher
SG	Cold ischemia (min)				1	1.03
	ALT		0.106	0.92	0.83	1.02
	Censior (preserv. solution)		0.237	0.13	0	3.87
ECD	Age (recipient)	34%	0.236	1.06	0.96	1.16
	MELD ≥ 20		0.035 *	10.94	1.18	101.22
	Cold ischemia (min)		0.049 *	0.99	0.99	1
	VAD		0.11	11.29	0.58	219.72
SG	Warm ischemia (min)	26.40%	0.404	0.97	0.9	1.04
	AST		0.477	1.04	0.94	1.15
ECD	MELD ≥ 20	28.90%	0.046 *	6.99	1.04	47.22
	TB		0.019 *	3.06	1.21	7.75
	CPA		0.043 *	17.84	1.1	291.08
SG	Age (recipient)	32%	0.076	1.15	0.99	1.35
	MELD ≥ 20		0.26	5.18	0.3	9.48
	OTI		0.194	2	0.7	5.72
	ALT		0.223	0.95	0.88	1.03
ECD	MELD ≥ 20	64%	0.014 *	10.81	1.62	72.16
	Cold ischemia (min)		0.154	1	0.99	1
	Trauma (cause)		0.26	3.72	0.38	36.5
	TB		0.022 *	7.11	1.33	37.93

OR - Odds-ratio; CI - Confidence interval; SG - Standard graft donor; ECD - Expanded criteria donor; OTI - Orotracheal intubation; AST - Aspartate amino transferase; ALT - Alanine aminotransferase; TB - Total bilirubin; VAD - Vasoactive drug; CPA - Cardiopulmonary arrest.

The logistic regression for the deaths during the evaluated period (0 to 90 days) did not indicate significance for any analyzed variable. In the high preoperative MELD SG group, we found a 5.18-fold increased risk of death, though not significant (p = 0.26). In the ECD group, the presence of elevated preoperative MELD was associated with a 10.81-fold increased risk of death (p = 0.014). In addition, the donor’s elevated total bilirubin (TB) was associated with an increase in the risk of death of 7.11 times (p = 0.022) (Table 3).

The occurrence of PNF and retransplantation was similar in both groups and the regression analysis did not show any risk or protection factor. 

## DISCUSSION

Since OLT was instituted as an excellence, definitive, and curative treatment for chronic and terminal liver diseases, there has been a progressive and continuous increase in waiting lists for transplantation. Therefore, alternatives were sought to increase organ supply. The first studies to try to identify factors related to worse results, such as advanced age, length of ICU stay, ischemia (warm and cold) time, laboratory tests, hemodynamic status, and use of VAD, date from 1993^17^ ^,^ ^20^.

In agreement with these data, we observed that 50.9% (56 cases) of the grafts used for transplantation during the study period were from ECD, which contributed to a significant increase in the number of transplants performed in this period due to the expansion of the liver grafts’ acceptance criteria.

Thus, the use of these ECD is, at the moment, one of the most widely used alternatives^21^ ^,^ ^22^. Several authors have demonstrated that these organs can be safely used when assessing the risk of operative complications compared with prolonged time on the waiting list for OLT, showing cost-effectiveness. Therefore, the vast majority of organs are used, discarding only those whose severe changes would render their utilization unethical^23^. The main objective of these publications was to demonstrate the factors that do not influence the result and those that can harm, according to the situation in which they are used.

Similar to the literature, when assessing overall survival (disregarding MELD), we observed slightly worse survival in the ECD group, but without statistical significance. However, when we compared it with studies that evaluate criteria in isolation, we saw similar results in the same way. For instance, in the SG group, we observed 100% survival in patients who received organs from donors aged 50 and over (six cases). However, in the ECD group, we found 62.5% survival in those patients who received organs from donors aged 50 years or older (eight cases). Therefore, regardless of the group, overall survival was 78.6% (three deaths in 14 cases), similar to the overall survival of 78.2% (24 deaths in 110 evaluated patients)^5^ ^-^ ^7^.

As for the MELD within each group, we found a slight difference in the groups with high MELD (≥ 20), especially in the ECD group. However, we only achieved statistical significance in the high MELD and ECD group, in which we observed an increase in mortality (up to 10.81 times), regardless of the period evaluated. This result is similar to the one of Bacchella et al., who found 64.7% 30-day survival in transplanted patients with high MELD who received ECD grafts, while those with low MELD and who received SG livers had a survival rate of 93.4%; we observed a survival rate of 73.3% and 95%, respectively, for this period^24^.

After implanting the MELD criterion for graft distribution, similar results were observed in 90-day survival (pre-MELD 93% vs. post-MELD 89%, ± 3), as observed by the Zurich group in Switzerland. These the authors found a significant decrease in mortality on the waiting list, from 25% in the pre-MELD era to 13% in the post-MELD era, in addition to the decreased time in the waiting queue, reduced from 334 to 204 days. However, with the increase in the MELD of patients undergoing transplantation and the consequent increase in the severity of the operated patients, although there is no worsening of survival, there was a significant increase in costs of approximately 55.5% per patient. These were due to the increase in the mean hospital stay and other factors^25^.

Among the factors used to assess the quality of the graft, the only one we found to be associated with increased mortality, both early and overall, was the increase in total bilirubin (> 2), with an increase in the risk of early death by 3.1 times, and overall death, by 7.1 times^26^.

Regarding the frequency of PNF, we found similar rates in both groups, similar to the literature reports^27^, but with rates of retransplantation lower than those published. Therefore, we could not associate PNF and retransplantation with the use of ECD organs.

Another important aspect when evaluating graft quality and possible solutions to increase organs supply is the assistance that potential donors receive when admitted or evaluated in hospitals^28^. Adequate and aggressive support should be given to every patient admitted or evaluated, even those with a poor prognosis, to provide the best care. However, in those cases in which serious injuries are identified that make it impossible for the patient to recover, one should identify potential donors as soon as possible and maintain recovery aggressively, even if ICU support, fluid resuscitation, use of VAD, and hormonal recovery, when indicated, are necessary to maintain their organs^28^ ^,^ ^29^. Thus, it is possible to decrease the loss of potential donors, improve quality, and decrease family refusal through teams engaged and prepared for such an approach, after adequate care for the victim^28^. 

We observed the importance of this aggressive support to maintain hemodynamic stability and prevent CPA. We identified that history of CPA, when associated with other risk factors for ECD graft malfunction, posed a higher risk of complications and mortality, increasing the risk of mortality by 17.9 times before 30 days, further justifying the importance of adequate donor care^28^ ^,^ ^30^.

Thus, in our survey, we observed the influence on survival of ECD receptors only by elevated bilirubin and a history of CPA, as well as worse results in the subgroup with high MELD score. In a way, this justifies the use of these organs. However, we must emphasize that this is a study with a relatively small sample and new studies with larger numbers of patients may reinforce the information found.

Ultimately, we observed that, in general, there is no optimal graft and the vast majority is utilized as long as there is no clear contraindication.

Expanded criteria donor grafts are widely used and can be safely utilized, with results similar to those of SG, both in the early period, in the late one, and overall. In those recipients with high MELD (≥ 20), the use of ECD livers has a negative impact on survival. Risk factors that negatively affect survival of ECD liver recipients are elevated bilirubin and CPA. There was no increase in PNF, nor a higher incidence of retransplantation, due to the use of ECD livers.

## CONCLUSION

Expanded criteria donor grafts can be safely used, with results similar to those of SG, both in the early period, in the late one, and overall. Risk factors that negatively impact the survival of ECD liver receptors are elevated bilirubin, CPA, and receptors with high MELD (≥ 20). There was no increase in PNF, nor a higher incidence of retransplantation, due to the use of ECD livers.
